# Oxidative Stress and the Homeodynamics of Iron Metabolism

**DOI:** 10.3390/biom5020808

**Published:** 2015-05-11

**Authors:** Nikolaus Bresgen, Peter M. Eckl

**Affiliations:** Department of Cell Biology, University of Salzburg, Hellbrunnerstrasse 34, 5020 Salzburg, Austria; E-Mail: peter.eckl@sbg.ac.at

**Keywords:** iron, oxidative stress, metabolism

## Abstract

Iron and oxygen share a delicate partnership since both are indispensable for survival, but if the partnership becomes inadequate, this may rapidly terminate life. Virtually all cell components are directly or indirectly affected by cellular iron metabolism, which represents a complex, redox-based machinery that is controlled by, and essential to, metabolic requirements. Under conditions of increased oxidative stress—*i.e.*, enhanced formation of reactive oxygen species (ROS)—however, this machinery may turn into a potential threat, the continued requirement for iron promoting adverse reactions such as the iron/H_2_O_2_-based formation of hydroxyl radicals, which exacerbate the initial pro-oxidant condition. This review will discuss the multifaceted homeodynamics of cellular iron management under normal conditions as well as in the context of oxidative stress.

## 1. Systemic and Cellular Iron Transfers

Ferric iron or iron contained in heme is absorbed by intestinal enterocytes via heme carrier proteins (HCP1) [1], the divalent metal transporter DMT1 (SLC11A2) [2,3] or the integrin-mobilferrin pathway [4,5] (a review on intestinal iron absorption is given in [6,7]). The absorbed iron is then released from the enterocytes to the bloodstream as transferrin-bound iron (TBI) via ferroportin (see below). Under physiological conditions, the bulk of iron enters the cell bound as TBI via transferrin-receptor (TfR) mediated endocytosis followed by endosomal iron liberation. However, resorption of non-transferrin bound iron (NTBI) from the bloodstream may also occur either via DMT-1, the zinc transporter Zip14 (SLC39A14) [8,9] or specific citrate binding sites [10,11,12]. Notably, the serum content of *labile* NTBI is very low under normal conditions but may rise substantially in diseased states, such as thalassemia, where the high NTBI level—essentially caused by repeated blood transfusion—is considered to cause disease-related oxidative stress [13,14,15,16,17,18]. Similarly, serum ferritin which may serve as iron carrier too [19,20] and is also increased under certain pathological conditions, such as inflammation and cancer [21], can also be endocytosed [22,23,24,25] upon binding to distinct ferritin receptors [26,27,28,29,30], TIM-2 [31,32], Scara5 [33] as well as the TfR itself [34]. Finally, heme-bound iron will enter the cells via HCP1 [1] and tissue macrophages will also “ingest iron” upon phagocytosis of aged cells such as erythrocytes or via the haptoglobin/CD 163 or hemopexin/CD91 mediated uptake of hemoglobin or heme [35] and deliver the recycled iron back to the bloodstream, which is indispensable for the maintenance of systemic iron homeostasis [36].

In contrast to several ways of cellular iron uptake, only two mechanisms of cellular iron release are known. Usually, iron release from a cell occurs via ferroportin (Fpn) [37,38,39,40,41] a membrane bound iron exporter, which is controlled by hepatocyte derived hepcidin [42,43], the hepcidin activity itself being regulated by the serine protease matriptase-2 [44,45]. The ferroportin-released iron is then directly transferred to transferrin by aid of the multi-copper ferroxidases hephaestin and caeruloplasmin [39,46,47,48]. Fpn-based iron release from enterocytes or macrophages is essential to systemic iron homeostasis, hepcidin acting as negative regulator of Fpn counteracting systemic iron overload [49]. Hypoxic conditions lower hepcidin expression and thus promote iron absorption [50,51], the negative regulation of hepcidin exerted by hypoxia inducible factor-1 [52] playing an important physiological role in the adaptation to increased altitudes [53]. Notably, apart from its role in systemic iron homeostasis, the Fpn-based iron release mimics the effect of iron chelators, such as desferrioxamine (DFO), by counteracting iron-based oxidative stress [54]. Supportive to this, reduced (or absent) ferroportin activity (e.g., upon hepcidin overexpression or mutation of the ferroportin gene) results in cellular iron overload [55,56]. Notably, mutation of the Fpn gene causes the so-called “ferroportin disease”—with symptoms of tissue iron overload reminiscent to hemochromatosis—however, at a less critical clinical manifestation [55]. In particular, unlike the hepatocytic iron overload seen in hemochromatosis, ferroportin disease patients show no hepatic iron accumulation. Noteworthy, serum ferritin levels increase in patients suffering from ferroportin disease [55], the secretion of iron loaded ferritin presumably protecting from hepatic iron overload.

Hence the relase of iron-loaded ferritin could represent a *non-orthodox* mechanism to avoid iron overload in cells that do not express Fpn or Fpn is inhibhited by high hepcidin levels which is also accompanied by increased serum ferritin levels [56]. The exact mechanism by which ferritin is secreted remains elusive, however, it has been shown that ferritin can be released via exocytosis in an iron dependent mode [57,58] and the release by secretory lysosomes has also been proposed [59]. Moreover, ferritin transcytosis has also been suggested [60]. Evidence exists that ferritin serves iron shuttling between cells including a presumptive role as iron transporter across “barriers” such as the blood-brain-barrier (BBB) or the placental brush border (PBB) [19,26,29,61,62]. The findings that uptake of extracellular ferritin may serve haemoglobin synthesis in erythroid precursor cells [24] and the use of ferritin (and not transferrin) as major iron source in oligodendrocytes [63] support this assumption. Furthermore, the ferritin content of serum correlates with total body iron stores [21,64] and is increasing upon dietary iron supplementation [65] and also with age [66,67]. In addition, several diseased states are accompanied by pathological changes of serum ferritin levels such as anemia-based hypoferritinemia [68,69] and the hyperferritinemia frequently associated with infection, inflammation and malignancy [21,64,68,70,71], which potentially complicates serum ferritin-based assessment of the body iron status [72]. Albeit this points at a role of extracellular ferritin in cellular and systemic iron homeodynamics and evidence is increasing for a participation of serum ferritins in systemic stress responses (see Section 2.2), our understanding of the biological significance of ferritin secretion and uptake still is incomplete.

## 2. Cellular Iron Compartmentalization

**Figure 1:**
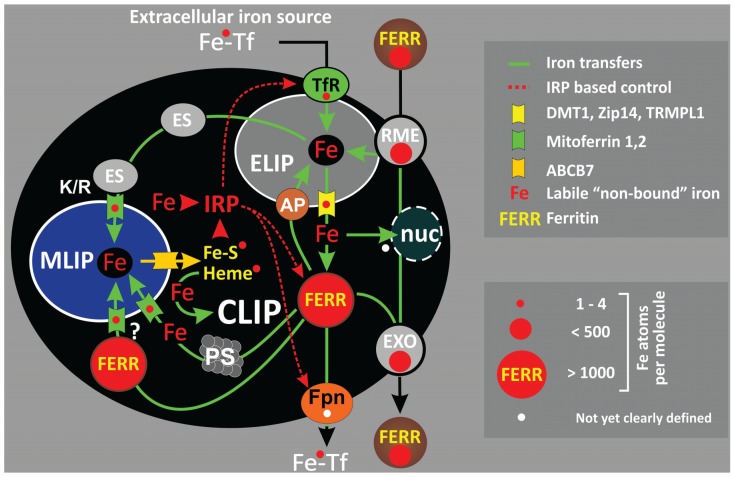
Cellular iron flux. Iron is transferred between communicating “labile iron pools” of the endo-/lysosomal system (ELIP), the cytosol (CLIP) and the mitochondria (MLIP). The ELIP represents the main entry site for extracellular iron such as transferrin bound iron (Fe-Tf) taken up via the TfR. Alternatively, iron containing serum ferritin may also enter the ELIP via receptor-mediated endocytosis (RME). Iron can exit from the ELIP via specific channels (DMT-1, Zip14, TRPML1) and is buffered in the cytosol (CLIP) by ferritin. Iron release from ferritin can occur via proteasomal degradation (PS) or lysosomal digestion upon autophagy (AP). Transfer of iron to the MLIP involves the iron transporter mitoferrin. The shuttling of “endosomal iron” (ES) to mitochondria by a “kiss and run” mechanism (K/R) as well as a hypothetical direct iron uptake from cytosolic ferritin has also been proposed. Note that iron can be buffered in the MLIP by mitochondrial ferritin. Ferroportin (Fpn) serves iron exit, the exact transfer mechanism not yet being resolved [39]. Fe-S clusters and heme, released from the mitochondria via ABCB7 transporter as well as labile, non-bound cytosolic iron serve as iron sensors for cytosolic IRPs. IRPs regulate the cellular labile iron pool via translational control of several iron-metabolism related proteins such as TfR, ferritin and ferroportin (Fpn). Alternative iron fluxes include heme-oxygenase 1 mediated iron liberation as well as ferritin endocytosis, transcytosis and exocytosis (EXO). The uptake of heme and extracellular NTBI is not shown. Red circles symbolize the relative iron binding capacities.

Metabolic requirements focus on proper iron supply for the mitochondrial synthesis of heme and iron-sulfur (Fe-S) clusters, functional groups which are indispensable for cell function and serve as central determinants of cellular iron “handling”. Conflicting with the strict demand for iron availability, free “labile”, redox-active ferrous iron is prone to generate highly reactive ^●^OH-radicals by reacting with H_2_O_2_ in the Fenton reaction eventually causing oxidative cell damage. Thus, intracellular iron is compartmentalized into distinct “cellular labile iron pools” which communicate via secure, protein-based iron shuttles (Figure 1).

Upon receptor-mediated endocytosis, iron will initially locate to the endo-/lysosomal compartment (ELC) from where it is forwarded to the cytosol via distinct iron transporters DMT-1, Zip14 or TRPML1 (mucolipin 1) [3,73,74,75,76]. With respect to the continuous need for iron, cytosolic ferritin will serve as dynamic iron buffer, which is essential to a steady-state of intracellular iron availability. Like ferroportin, ferritin will also counterbalance a transient iron overload by sequestering an excess of Fe^2+^ and thus confer antioxidant and cytoprotective functions [77,78]. However, most of the imported iron will be delivered immediately to the “users”, in particular mitochondria, which may involve a bypass of cytosolic iron buffering (see below).

About 0.2%–5% of the total cellular iron is considered as transiently mobile, non-protein bound low molecular weight redox-active iron which together with chelatable protein-bound iron defines the dynamic, intracellular “labile” iron pool (LIP) encompassing compartment specific LIPs of the cytosol (CLIP—also including nuclear labile iron), the mitochondria (MLIP) and the endo-/lysosomal compartment (ELIP) in total containing about 6–16 µM iron, mainly as Fe^2+^ [79,80,81,82,83,84]. Iron is shuttled between these pools by several distinct mechanisms: (i) distinct donor–acceptor exchanges (e.g., iron uptake and release to and from ferritin); (ii) transfer of iron across membranes by iron transporters such as DMT-1, TRPML1, Zip14 (ELIP → CLIP); and (iii) iron “binding” to mitoferrin, paraferritin (see below) (CLIP → MLIP) and ferritin (CLIP → ELIP via autophagy) [79,85,86]. As mentioned, endosomal iron (ELIP) may also be transferred directly to mitochondria by a “*kiss and run*” mechanism, iron containing endosomes or iron containing vesicles docking to the outer mitochondrial membrane and passing the iron over to mitoferrin [87,88,89,90,91]. Notably, the latter mechanism circumvents the CLIP and allows an efficient direct transfer of iron to mitochondria which may be beneficial under physiologic conditions but could become critical under conditions of iron overload [90]. Transfers involving free ferrous iron represent a constant hazard of Fenton-reaction derived oxidative stress, which holds particularly true under conditions of iron overload and increased oxidative stress.

Lysosomal processing of iron loaded ferritin either upon autophagy or heterophagic uptake of “serum ferritin” represents such a situation where adequate coupling of the liberated ferrous iron to adequate acceptors is mandatory to avoid an excess of redox-active iron in the lysosomes. It has been shown that autophagy of apoferritin protects lysosomes from iron overload and exerts cytoprotective properties [92,93,94]. On the other hand, ferritin heterophagy may stimulate lysosomal stress and resulting growth adverse responses [95,96].

### 2.1. Endo-/Lysosomal Iron—ELIP

Intestinal iron absorption as by enterocytes occurs separately for Fe^2+^ and Fe^3+^ via the divalent iron transporter DMT-1, which also serves the uptake of ferrous NTBI in other cell types and the β3-integrin-mobilferrin pathway [5]. β3-integrin forms a large protein complex together with mobilferrin also incorporating DMT-1, which shows ferrireductase activity, and due to its size has been named paraferritin [97,98]. Intracellular paraferritin is considered to serve the shuttling of iron to mitochondria, which may also apply to iron derived from transferrin [6]. Different from this, iron adsorption of TBI occurs via TfR mediated endocytosis, the transferrin/TfR complex locating to the endosomal compartment where the acidic milieu supports the release of transferrin bound Fe^3+^. The iron-free apotransferrin/TfR complex is then recycled to the cell membrane where it dissociates, both components serving further iron acquisition. Inside the acidified endo-/lysosomal compartment, the liberated ferric iron is reduced to the ferrous state by the endosomal ferrireductase Steap3 [99,100], which is highly expressed in iron-rich cells, such as hepatocytes and macrophages [101]. The ferrous iron is then released from the endosomal compartment to the cytosol either via DMT-1 or the lysosomal iron channel TRPML1, a type IV mucolipidosis-associated protein also termed mucolipin-1 [73,76]. Interestingly, impaired TRPML1 function provokes a severe disturbance of cellular iron homeostasis, marked by cytosolic Fe^2+^ depletion and lysosomal Fe^2+^ overload, accompanied by the lysosomal accumulation of the indigestible lipid-protein oxidation product lipofuscin, sensitizing lysosomes to oxidative stress, which is causal to hereditary mucolipidosis and other lysosomal storage disorders involved in neurodegenerative diseases [73,102,103,104,105].

Notably, compared to transferrin which provides two iron ions (Fe^3+^) per molecule, the uptake of serum ferritin via receptor mediated endocytosis expands the lysosomal labile iron pool more substantially since serum ferritins, albeit considered iron poor, may contain about 160–500 Fe^3+^ ions per molecule [106,107]. Moreover, TfR-based iron import is regulated by cellular iron concentrations while this is not the case for the uptake of ferritin via asialoglycoprotein receptors [108]. Besides iron import from the environment, the lysosomal iron pool may also expand upon organelle recycling by macroautophagy of iron-rich mitochondria (mitophagy) and peroxisomes (pexophagy) as well as lysosome recycling itself [109,110,111,112], which represents a particularly critical issue during damage induced reparative autophagy [113,114]. Furthermore, macroautophagy of cytosolic iron-loaded ferritin and iron liberation by lysosomal processing will also expand the ELIP [115].

With respect to the different iron input routes feeding the ELIP, iron release from the lysosome is pivotal to the maintenance of proper levels of lysosomal labile iron. Moreover, the pathogenic effect of TRPML1 failure emphasizes the necessity for a stringent ELIP control, especially in post-mitotic cells such as neurons. Several reports have shown the dependence of lysosomal stability on lysosomal iron load [92,94,116,117,118,119,120]. Critical to this, the Fe^2+^-based ^●^OH formation is fostered by pro-oxidant conditions, which stimulates lysosomal lipid peroxidation and consequently increases the susceptibility to lysosomal membrane permeability (LMP) and subsequent cell death [117,118,121,122,123,124,125,126,127,128]. Evidence is increasing that cells are able to antagonize lysosomal iron overload and the oxidative stress derived thereof by transferring antioxidants, including apoferritin, to the ELC by macroautophagy [93,94,129]. Moreover, the pro-oxidant conditions arising from LMP have a stimulatory effect on *de novo* ferritin synthesis [94,130]. Summarizing, the dynamics of the ELIP serve as “rheostat” of cellular iron flux, which in context with the pro-/antioxidant balance couples redox control to lysosome stability and cell integrity.

### 2.2. Ferritin—CLIP

As stated above, ferritin-based iron-buffering is crucial to cellular integrity in particular under conditions of increased oxidative stress. Albeit ferritin has a maximum storage capacity of 4500 Fe^3+^ ions per ferritin molecule [78], biological ferritin samples may contain less iron (<2000 Fe^3+^/ferritin molecule), which holds particularly true for secreted, serum ferritins as already stated [106,107,131]. Ferritin is a multimeric protein with a molecular weight of about 450 kDa, composed of 24 heavy (H chain of 21–23 kDa) and light (L chain of 19–21 kDa) subunits arranged in a hollow sphere conformation [132]. Several tissues-specific isoforms have been described which vary in the H:L chain ratio and with respect to their pI are classified as basic, L-rich (e.g., liver, spleen) or acidic, H-rich (e.g., muscle, heart, brain) isoferritins [132,133,134].

Contrasting the low sequence homology (in mammalians about ~54%), the ferritin L and H chain show a remarkably high conformational homology [132]. Iron enters the ferritin molecule in the ferrous state via three-fold channels. The entrance is facilitated by the chaperone PCBP1 (Poly-r(C)-Binding Protein 1) [135] and involves oxidation by the ferroxidase activity of the H-chain, a process which also consumes H_2_O_2_ [132,136]. The oxidized iron is then shuttled to the inner cavity of the ferritin molecule where it is stored in a mineralized form, the nucleation process aided by the L-chain [137,138]. Iron exit from ferritin (recently reviewed in [139]) involves the reduction of the mineralized Fe^3+^, however, may also involve electron transfers, gated pores as well as direct iron release mechanisms, which are not based on reduction (reviewed in [140]). Small reducing molecules such as O_2_^●−^, ascorbate or the ascorbate radical [132] and NO^●^ (see below), but also sulfide [141] and hydroxydopamine (6-OHDA) [142] are able to mobilize iron from intact ferritin *in vitro*. Ionizing radiation together with ascorbate may also trigger O_2_^●−^-dependent iron release which expands the LIP in *post mortem* tumor tissue [143]. Ferritin degradation appears to play an important role in iron release from ferritin *in vivo* [144,145,146,147], which can be stimulated by iron chelators [148] and oxidative stress [149]. Notably, while iron chelators stimulate autophagy and lysosomal ferritin processing, iron release triggered by ferroportin precedes proteasomal digest of the “iron-depleted” ferritin [42,145,148]. Thus, iron liberation from ferritin may follow different context-dependent routes serving either cellular iron release (proteasome) or the refueling of a rapidly emptied CLIP (autophagy/ELC). Considering the above discussed risk of lysosomal destabilization arising from ferritin autophagy in oxidatively stressed cells, CLIP depletion under prooxidant conditions will become a potent, cytotoxic challenge when the cell is not adequately equipped with antioxidants including newly synthesized apoferritin.

Emphasizing the antioxidant properties of ferritin, overexpression of human H-chain ferritin confers solid protection against oxidative stress [136]. It has been shown that iron mediated lipid peroxidation is suppressed by recombinant H and L ferritins *in vitro*, which requires both the ferroxidase as well as iron mineralization activity [150]. While L-chain rich isoforms stably incorporate iron at increased cellular iron levels [151], the higher ferroxidase activity of H chain-rich ferritins allows a faster and more efficient iron sequestration, which also improves the antioxidant and cyto-protective potential under conditions of oxidative stress [78,132,152]. Thus, with respect to the coordinated regulation of iron oxidation and iron storage, the ferritin H:L ratio plays an important role in tissue specific iron regulation and antioxidant defenses [152], which probably affects the aging process too since the life-span in *Saccharomyces cerevisiae* and *Caenorhabditis elegans* is extended upon (over)expression of the human ferritin L-chain [153].

Cellular ferritin levels are regulated primarily at the translational level via m-RNA binding proteins IRP-1 and IRP-2 described below. Nevertheless, the genes for the ferritin H and L chain contain binding sites for NF-κB/Rel and elements with similarity to AP-1, which mediate transcriptional control of the H-chain by the oncogenes c-Jun and c-Fos [154,155]. Moreover, antioxidant response elements (ARE) located in the promoter region of both ferritin genes allow binding of Nrf2 and junD [156,157,158], which also links transcriptional control of ferritin to cellular stress management. Thus, ferritin synthesis can be regulated in an iron independent mode for instance by inflammatory cytokines (e.g., TNF-α, IFNγ, IL-1β, IL-6—preferentially stimulating H-chain expression) and oxidative stress, which confers cytoprotection [58,159,160,161,162,163,164,165,166,167,168,169]. Strikingly, p53 affects ferritin synthesis too either as negative regulator on the transcriptional level supposedly weakening cellular antioxidant defense in favor of promoting apoptosis [170] or by upregulating ferritin biosynthesis which supports cell survival due to an increased iron sequestration [171]. In addition, experimental evidence exists that the iron controlled synthesis of the ferritin H and L chain occurs independently of each other and is modulated by cellular oxygen levels at the transcriptional as well as translational level [172,173,174,175,176]. Hence, ferritin synthesis is regulated by multiple elements of transcriptional and translational control, which supports a tight, dynamic linkage of iron buffering and the oxidant balance to cell cycle and cell death control.

The stimulation of ferritin synthesis by inflammatory cytokines can be paralleled by enhanced ferritin secretion [58]. In accordance with this, serum ferritin levels are increased in inflammatory contexts rendering ferritin an acute phase reactant [177,178,179,180,181]. It is assumed that this elevation of serum ferritin counteracts iron mediated oxidative stress in the inflamed tissue and, in the case of bacterial infection, probably also limits iron availability for bacterial growth [159,177,178,180,182]. Since proinflammatory cytokines preferentially promote ferritin H-chain expression [169], acute phase associated serum ferritins likely represent H-chain rich isoforms [180] which differ from the L-type ferritin found in serum under normal conditions [21]. Therefore, the inflammation related switch towards H chain-rich serum ferritins which reveal an improved antioxidant activity corresponds well with the enhanced synthesis of H chain-rich, cyto-protective ferritin in oxidatively stressed cells [136,173]. Hence, similar to ferritin mediated iron buffering in the CLIP, ferritin secretion may expand systemic iron buffering capacity in particular under pro-oxidant conditions.

Finally, it should not be neglected that heme-containing proteins, despite the minimal iron binding capacity of protoporphyrin, also affect cellular iron homeodynamics. While heme-biosynthesis lowers the MLIP (discussed in the next section) [183], heme-degradation by heme-oxygenase I (HO-I) [184] potentially adds iron to the CLIP which may occur in the cytosol but alternatively could also locate to the ELIP in macrophages following erythrocyte phagocytosis [185,186]. Free heme sensitizes cells for cell death especially under pro-inflammatory, pro-oxidant conditions in an iron dependent mode [187,188,189]. Thus, heme degradation by HO-I will confer cyto-protection provided the released iron is efficiently sequestered by ferritin [190]. In line with this, HO-I and ferritin are concomitantly upregulated in cells exposed to oxidative stress [191,192], and cytosolic iron overload caused by an excessive, HO-I-based heme-degradation such as seen in malaria may also be antagonized by the up-regulation of H-ferritin [193].

### 2.3. The Mitochondrial Iron Pool—MLIP

Complementary to ELC and ferritin-based iron handling, proper mitochondrial iron homeostasis is indispensable for cellular iron management in particular by controlling the synthesis of heme and Fe-S clusters [194,195,196,197], functional groups which are essential to the functionality of numerous proteins including those participating in energy production via the respiratory chain. Intimately connected with this, the MLIP also affects the synthesis of Fe-S cluster containing iron regulatory protein-1 (IRP-1) and by this directly interferes with IRP-based translational control of proteins involved in iron management, in particular ferritin, the transferrin receptor, ferroportin and DMT-1. Thus, the iron flux between the CLIP and the MLIP has to be tightly coordinated and is also regulated by a feedback mechanism based on Fe-S cluster synthesis and IRP-1 activity. The MLIP is fueled from the CLIP as well as directly from the ELIP as explained above. Albeit the knowledge on iron shuttling across the outer mitochondrial membrane is still incomplete, the endosomal supply via a “kiss and run” mechanism could involve a distinct endosome-mitochondria interaction and the existence of specific mitochondrial ferritin binding sites has been proposed too [85,198]. In contrast, it has been demonstrated that the iron transfer across the inner mitochondrial membrane is mediated by the mitochondrial iron carrier mitoferrin 1,2 (Mfr1,2) [87,199,200], which may involve the regulation via Mfr protein stability as suggested by the interaction of Mfr1 with the *mitochondrial inner membrane ATP-binding cassette transporter* Abcb19 in erythroblasts [201]. Notably, Mfr1 and 2 are not regulated by IRPs but other *cis*-acting elements [202]. Whatever mechanism is responsible for mitochondrial iron uptake, it has to be strictly controlled since any disturbance of the MLIP will critically affect cellular iron management. This is exemplified by pathological conditions caused by mitochondrial iron mismanagement including Friedreich’s Ataxia (caused by frataxin deficiency—see below), erythropoietic protoporphyria (a disorder marked by ferrochelatase deficiency and impaired heme synthesis) or sideroblastic anemia (where disrupted heme synthesis alters mitochondrial ferritin levels, the enriched mitochondria resulting in ringed sideroblasts) reviewed in [203,204,205,206].

Of special relevance, an expansion of the MLIP will also occur when Fe-S clusters (e.g., contained in mitochondrial dehydratases such as aconitase) are oxidized by superoxide (O_2_^●−^) formed upon electron leakage from the respiratory chain (reviewed in [207]). Moreover, albeit mitochondrial superoxide dismutase (MnSOD) will detoxify O_2_^●^, the dismutase reaction will generate H_2_O_2_, which readily reacts with “labile” ferrous iron leading to the generation of ^●^OH radicals and subsequent lipid peroxidation in the mitochondrial compartment. Notably, cytochrome c oxidase which catalyzes the electron transfer in complex IV of the respiratory chain is inhibited by the lipid peroxidation product 4-hydroxy-nonenal (HNE) [208]. Thus an expanded MLIP at inadequate antioxidant defenses will promote mitochondrial damage with severe consequences. Indeed, Halliwell (1992) has pointed at this adverse effect of SOD-activity, which may occur under certain pathologic conditions [209]. Therefore, the MLIP has to be maintained at an optimum balance which sufficiently serves metabolic needs but does not “fuel” Fenton reaction-based oxidative damage. Moreover, degradation of iron overloaded, damaged mitochondria in autophagolysosomes will amplify the effect of mitochondrial oxidative stress by stimulating lysosomal iron overload as illustrated above [210,211].

It has to be noted that no distinct iron exporter has been identified in mitochondria, the release of newly synthesized heme and Fe-S clusters to the cytosol representing the only way that the MLIP can be lowered. Fe-S clusters are transferred by mitochondrial ATP-binding cassette proteins Atm1, ABC protein 3 and ABCB7 (the human ortholog of yeast Atm1), which has been shown to counteract mitochondrial iron overload [212,213,214,215]. Correspondingly, deficiency of such proteins will increase the MLIP, which has been shown for yeast Atm1 raising the mitochondrial iron content by about 30-fold [216] and also holds true for ABCB7 deficiency in humans causing the rare hereditary disease X-linked sideroblastic anemia and ataxia (XLSA/A) [214,217]. The way that heme is transported to the cytosol remains to be clarified; however, the involvement of a heme-carrier protein, such as heme binding protein-1 (p22 HBP), has been suggested [87,218]. The lack of distinct iron exporters emphasizes the mitochondrial compartment as “bottleneck in iron metabolism” the incoming iron being either directly consumed for Fe-S cluster- and heme synthesis or stored in mitochondria-resident proteins, such as mitochondrial ferritin (see below).

### 2.4. Mitochondrial Iron Usage—Frataxin and Mitochondrial Ferritin

Frataxin (Ftx; Yfh1p in yeast; CyaY in bacteria; reviewed in [219,220]) represents an iron binding protein of high relevance to mitochondrial function and integrity. Ftx can form multimeric complexes of different size, which can bind between 50–3000 iron ions (depending on the species and grade of multimerization) in its ferric state, although the iron binding capacity of Ftx may be much lower *in vivo* [221,222]. Opposite to ferritin, which is synthesized as iron-free apoferritin, assembly of Ftx multimers depends on iron [221].

Ftx has gained substantial interest over the last decade since mutations of the Ftx gene are causal to the autosomal recessive disease Friedreich’s Ataxia (FRDA) [205,206,223,224], most patients showing strongly reduced levels of Ftx mRNA [223,225] and protein [226]. The disease is marked by severe neurological manifestation, as well as pathological changes of the skeleton (scoliosis), pancreas (diabetes mellitus) and heart. In fact, cardiomyopathy and cachexia represent the most frequent cause of death in FRDA patients; for a detailed review on FRDA see [205] and recent advances in Ftx research are compiled in [219]. From the biochemical point of view, FRDA is marked by iron accumulation and lipofuscin deposits [227]. In particular, FRDA is accompanied by a reduced content of mitochondrial Fe-S cluster containing proteins and a loss of aconitase activity [228], which points at the primary function of Ftx, acting as “iron chaperone” in providing iron for the scaffold protein ISCU (iron-sulfur cluster forming unit) which is essential to mitochondrial Fe-S cluster biosynthesis [229,230,231]. Ftx also serves the transfer of iron to mitochondrial membrane associated ferrochelatase [232], a Fe-S cluster containing protein which catalyzes the final step of heme biosynthesis, the transfer of iron to protoporphyrin IX [233,234].

Albeit Ftx primarily serves ISCU formation, further functions include mitochondrial iron trafficking as well as mitochondrial redox and ROS control [87,235]. Ftx-deletion in fibroblasts yields a characteristic cellular FRDA phenotype, including mitochondrial iron deposits, reduced Fe-S enzyme activity and degenerating mitochondria [229]. Also, neurons of Prp-CreER^T^ mice, a mouse model for FRDA, show aberrant autophagy, large vacuoles and lipofuscin accumulation [236]. Notably, malfunctioning mitochondria are recycled by mitophagy, which promotes lipofuscinogenesis and renders lysosomes unstable when occurring in excess [237,238]. Hence, Ftx-deficiency may also hamper ELC function and the autophagic process. Pointing at secondary effects of Ftx deficiency in FRDA, markers for lipid peroxidation (malondialdehyde) and oxidative damage (8-hydroxy-2'-deoxyguanosine) are increased in urine and blood of FRDA patients [239,240,241]. This indicates the onset of iron derived, free radical-based oxidant events upon loss of Ftx activity which may be detrimental to mitochondrial function and will also affect the whole cell. In fact, Ftx deficiency can promote ROS generation in mitochondria (*i.e.*, formation of ^●^OH by the Fenton reaction), which is accompanied by oxidative mitochondrial damage and the upregulation of ferritin gene expression via nitric oxide (NO) signaling [242,243]. Furthermore, Ftx deficiency also sensitizes cells to oxidative stress [239,244], which may involve both mitochondria and the ELC as discussed above. Taken together, Ftx represents an important regulator of mitochondrial and cellular iron homeodynamics in particular under conditions of iron overload and oxidative stress. It is tempting to consider Ftx as mitochondrial iron buffer, which similar to cytosolic ferritin provides “antioxidant” properties. However, different from the yeast Ftx knock-out mutant ΔYfh1 [245] and Ftx-deleted mammalian cells, mitochondrial iron deposits have neither been found in FRDA patients [246] nor in Ftx-deficient Prp-CreER^T^ mice [247]. Also, Ftx oligomerization is not critical to Ftx function *in vivo* [229]. Hence, Ftx may not serve MLIP iron buffering *in vivo* [246,248], but support cell integrity and iron homeodynamics by its participation in ISCU/Fe-S assembly for IRP-1 synthesis.

Another mitochondrial iron binding protein—mitochondrial ferritin (mtFER)—was discovered about a decade ago [249] (reviewed in [250]). mtFer, encoded by an intronless gene shows homology to H-ferritin and also bears a ferroxidase center [251]. Albeit mtFER ferroxidase activity and iron uptake kinetics show some differences to cytosolic ferritins, mtFER is also arranged as 24-subunit homopolymer, which efficiently oxidizes and sequesters ferrous iron [252,253]. In contrast to cytosolic ferritin, however, translation of mtFER is not under the control of iron since the mtFER-mRNA lacks iron regulatory elements (IRE), the 5' UTR being mutated to a leader sequence which mediates mitochondrial targeting [249]. Also different from cytosolic ferritin, mtFER-mRNA has been found at high abundance only in testis and spermatozoa, lower amounts in the brain, kidneys, pancreas (islets of Langerhans) and thymus but is absent from tissues with high iron storage function such as liver and spleen [249,251,254]. Of pathological relevance, mtFER is increased in erythroblast mitochondria of patients suffering from sideroblastic anemia [203] an erythrocyte phenotype (ring sideroblasts), which is also found in XLSA/A patients, caused by ABCB7 deficiency-based mitochondrial iron accumulation (see above). Although the exact role of mtFER in iron metabolism remains to be defined in detail it has been shown that overexpression of mtFER in tumor cells leads to iron relocation from the cytosol to the mitochondria provoking mitochondrial iron accumulation and cytosolic iron depletion which abrogates ferritin synthesis and stimulates TfR production [254,255,256]. Overexpression of mtFer in tumor cells also modulates cellular ROS levels and—like Ftx deficiency—increases the sensitivity to oxidative stress leading to the onset of apoptosis. Notably, this “toxic” effect of mtFER overexpression is considered to result from increased lysosomal degradation of iron overloaded mitochondria which leads to a shift of redox-active iron in the lysosomes, oxidative stress and the enhanced consumption of antioxidants [257,258]. Furthermore, mtFER as well as cytosolic ferritin containing deposits are found in mitochondria of Ftx-deficient cardiomyocytes of FRDA patients, which points at the role of iron/ferritin derived mitochondrial damage in cardiomyocyte cell death [259]. On the contrary, enhanced expression of mtFER may also rescue Yfh1 deficient yeast cells as well as Ftx deficient Hela cells from mitochondrial dysfunction and confer protection from iron mediated oxidative injury [260,261,262].

Based on this, it is obvious that mitochondrial iron binding proteins by representing “guardians” of the MLIP provide an essential control of general, cellular integrity. It cannot be ruled out that endocytosed ferritin is transferred to the ELC and ferritin containing endosomes may also directly attach to mitochondria according to a “kiss and run” mechanism providing iron to the mitochondria as suggested by Ulvik [85]. If so, the interaction of internalized ferritin with mitochondria would likely result in the same outcome as seen upon mtFER overexpression: enhanced oxidative stress and free radical-based organelle damage, which eventually triggers apoptotic cell death. 

## 3. Iron Regulatory Proteins (IRP-1, IRP-2)

### 3.1. IRP-1: Redox-Based Control of Cellular Iron Homeodynamics

Several proteins which show redox-based properties contain Fe-S clusters of the types [4Fe-4S], [3Fe-4S] and [2Fe-2S]. For example, these include ferrochelatase, ferredoxin (providing electron transfer for cytochrome P-450 activity), aldehyde oxidase 1 (AOX1), xanthine dehydrogenase/xanthine oxidase, glutaredoxin 2 (a GSH-dependent oxidoreductase), nuclear proteins involved in DNA and RNA metabolism and DNA repair, and proteins of the mitochondrial respiratory chain participating in the assembly of the electrochemical gradient [263] (a recent review on the sensory and regulatory functions of Fe-S proteins is given in [264]). Of special relevance, Fe-S cluster bearing iron regulatory protein 1 (IRP-1) exerts a dual function. In the presence of “iron replete” [4Fe-4S] clusters, IRP-1 shows cytosolic aconitase activity (c-aconitase; ACO1) catalyzing the citrate to isocitrate interconversion in the cytosol, which regulates cellular NADPH levels [265,266]. However, low cytosolic iron levels as well as pro-oxidant regimens promote cluster disassembly, which abolishes c-aconitase activity but uncovers IRP-1 mRNA binding properties [265]. IRP-1 binds with high affinity to IREs marked by a stem-loop located at the 5' and 3' UTR of mRNAs encoding iron-regulatory proteins, in particular ferritin and Fpn (5' UTR located IRE) as well as TfR, DMT-1 and eALAS (erythroid 5-aminolevulinate synthase producing the heme precursor 5-aminolaevulinic acid) with an IRE at the 3’ UTR [267,268]. Hence, the Fe-S cluster dependent control of IRP-1 activity serves as redox-active iron sensor which links translational control to cellular iron homeodynamics. Importantly, the Fe-S cluster-based IRP-1/IRE interaction either represses or induces translation of the target mRNAs, thus allowing a precise, efficient control of intracellular iron fluxes: CLIP depletion will favor cluster disassembly and promote IRP-1 RNA binding which inhibits ferritin (and ferroportin) synthesis but triggers TfR (and DMT-1) synthesis resulting in enhanced iron uptake and limited iron sequestration [269]. At increased iron levels, IRP-1 RNA binding activity declines, the IRE release allowing enhanced iron buffering and limited iron influx.

Hence, the continuous control of cellular iron fluxes by the specific IRP-1 activity participates in ELIP, CLIP, and indirectly also MLIP iron balance. Notably, the fact that Fe-S clusters are synthesized in the mitochondria emphasizes the significance of the MLIP to cellular iron homeodynamics. Indeed, Fe-S clusters may report iron loading of the MLIP since a hindrance of Fe-S cluster synthesis due to ISCU inactivation excessively shifts iron transfer to mitochondria which depletes the CLIP and increases IRP-1 RNA binding activity resulting in a disturbance of cellular iron homeodynamics [270].

### 3.2. Fe-S Cluster Oxidation

IRP-1 activity is modulated by ROS, which modify Fe-S cluster conformation, including cluster destabilization, and can also lead to IRP-1 degradation, connecting iron management and metabolic activity to cellular ROS production [271,272,273,274,275]. Oxidation of [4Fe-4S] clusters by O_2_^●−^and H_2_O_2_ yields the [3Fe-4S]-IRP-1 conformation, which lacks aconitase activity (Equations (1) and (3)), the reuptake of Fe^2+^ restoring the [4Fe-4S]-IRP conformation (Equation (4)) and aconitase activity, which is supported by sulfhydryls such as glutathione (GSH) (Equation (5)) [273,276,277,278,279].

[4Fe-4S]^2+^ + **O_2_**^●**−**^ + 2H^+^ → [3Fe-4S]^+^ + **Fe^2+^ + H_2_O_2_** (1)

[4Fe-4S]^2+^ + **H_2_O_2_** → [4Fe-4S]^3+^ + OH**^−^ +^●^OH** (2)

[4Fe-4S]^3+^ → [3Fe-4S]^+^ + **Fe^2+^** (3)

[3Fe-4S]^+^ + e^−^ → [3Fe-4S]^°^ + Fe^2+^ → [4Fe-4S]^2+^ (4)

[3Fe-4S]^+^ + Fe^2+^+ GSH → [4Fe-4S]^2+^ + ½ GSSG + H^+^ (5)

[4Fe-4S]^2+^ + **H_2_O_2_** → **[4Fe-4S/O]^2+^** + H_2_O (6a)

[4Fe-4S/O]^2+^ + H^+^→ [3Fe-4S]^+^ + Fe^3+^ + OH**^−^** (6b)

Fe^2+^ + **H_2_O_2_** → Fe^3+^ + OH**^−^** +**^●^OH** (7)

Notably, albeit cluster oxidation by H_2_O_2_ could theoretically generate ^●^OH radicals (Equation (2)), it is more likely that the oxidation of [4Fe-4S] by H2O2 leads to ferryl-radical [4Fe-4S/O]2+ clusters (Equation (6a)) from which [3Fe-4S]+ clusters are derived (Equation (6b)) [277]. Nevertheless, Fe^2+^ and H_2_O_2_ represent harmful byproducts of Fe-S cluster oxidation (Equations (1) and (3)) which may generate ^●^OH radicals in the Fenton reaction (Equation (7)). Indeed, Fe-S oxidation-based ^●^OH radical formation represents a potent killing mechanism in bacteria, which is supposed to underlie the H_2_O_2_-based antimicrobial defenses used by higher organisms [257,277]. Moreover, inactivation of mitochondrial aconitase by O_2_^●−^ mediated Fe-S cluster oxidation causes necrotic cell death of embryonic rat cortical cells [276] which may be connected with the interruption of energy metabolism (mitochondrial aconitase—ACO2—is a key enzyme of the TCA cycle) and Fenton-reaction-based ^●^OH formation as stated above. With respect to the abundance of Fe-S cluster containing proteins of the respiratory chain, enhanced ^●^OH formation in fact could be of considerable relevance since it may directly exert protein damage and also stimulate lipid peroxidation (LPO), aldehydic LPO metabolites such as malondialdehyde or HNE leading to mitochondrial malfunction, instability and cellular collapse [208,280,281,282,283,284,285,286]. For instance, HNE may form adducts with cysteine residues of the cubane Fe-S cluster and the catalytic center of ACO2 which substantially lowers the enzymatic activity [287] and could also interfere with the RNA binding properties.

Moderate cytosolic Fe-S cluster oxidation changing the cluster conformation to the [3Fe-4S] state will abolish IRP-1 c-aconitase activity but this not necessarily is sufficient to induce IRP-1 mRNA binding activity [288]. Enhanced pro-oxidant conditions, however, will stimulate cluster decomposition, which promotes IRP-1 RNA binding, an effect which is of particular relevance to iron metabolism in cultured cells exposed to increased, non-physiologic oxygen concentrations [265]. Interestingly, IRP-1 RNA binding is stimulated by extracellular H_2_O_2_ while the endogenous, cytosolic H_2_O_2_ production shows no comparable effect [289,290]. This suggests the involvement of additional, Fe-S cluster independent, mechanisms controlling IRP-1 activity. Indeed, IRP-1 contains a phosphorylation site for protein kinase C [291,292], which allows an integration of IRP-1 activity in cellular stress responses. Moreover, phosphorylation of IRP-1 sensitizes for iron-dependent protein degradation and by this controls IRP-1 abundance *per se* [293]. Similarly, enhanced oxidative stress and massive iron overload also facilitate IRP-1 degradation, which will enhance iron buffering by altered ferritin synthesis at a limited TfR-based iron uptake [294,295,296]. Albeit this suggests that ROS triggered IRP-1 degradation acts as “emergency break”, limiting the labile iron pool under pro-oxidant conditions [267,275,297], IRP-1 degradation not necessarily changes intracellular iron levels [298]. Thus, under conditions of increased oxidative stress, the labile iron pool may be controlled by more than a single IRP-1-based mechanism.

Hence, IRP-1 apparently regulates iron homeodynamics at rather extreme conditions. While IRP-1 degradation restricts the labile iron pool at elevated ROS concentrations, IRP-1 RNA-binding counteracts iron-depletion and stabilizes the LIP under moderately increased oxidative stress, both mechanisms also controllable by additional cellular stress signals via IRP-1 phosphorylation. However, it should not be ignored that stimulation of IRP-1 RNA-binding by moderate ROS attack may also occur under iron-replete conditions which may promote cellular iron overload. Concerning the ELC/ELIP, this inappropriate response is prone to generate lysosomal stress, which alters the susceptibility for lysosome mediated cell death under pro-oxidant conditions [299,300]. Complicating the issue, inactivation of IRP-1 c-aconitase activity by Fe-S cluster oxidation will also weaken antioxidant defenses since IRP-1 c-aconitase activity contributes to both glutathione (GSH) synthesis and NADPH generation which is necessary for the reduction of oxidized glutathione (GSSG) [301,302,303,304]. Thus, a cytotoxic condition can readily emerge from mild oxidative stress if oxidation of IRP-1 Fe-S clusters leads to an inadequate disturbance of iron homeodynamics and antioxidant defenses.

### 3.3. IRP-2

Similar to IRP-1, iron regulatory protein 2 (IRP-2) also shows RNA-binding properties, however, lacks Fe-S clusters as well as aconitase activity and is regulated via proteasome—mediated degradation [265,305,306,307,308,309]. Among several target mRNAs, IRP-2 shows a preference to bind to ferritin H and L chain mRNA which is stabilized by proteasome inhibitors abrogating ferritin synthesis while iron-rich conditions promote proteasomal decomposition of IRP-2 [310,311,312]. Hypoxia stabilizes IRP-2 RNA binding too, which is also antagonized by iron [307,313,314]. Importantly, at physiologic oxygen concentrations (3%) IRP control of cellular iron levels is mainly exerted by IRP-2, IRP-1 showing little mRNA binding activity and marginal iron responsiveness [314,315]. On the contrary, at increased tissue oxygen tensions, IRP-2 abundance declines and IRP-1 adopts the role as main iron regulatory protein [314] as discussed above. IRP-2 RNA binding is upregulated by phosphorylation, which, different from IRP-1, is iron dependent and does not increase IRP degradation [293,316]. However, similar to IRP-1, phosphorylation links IRP-2 activity to intra- and extracellular signaling which may serve cell proliferation and differentiation [316]. For instance, it has been shown that IRP-2 couples Jak/Stat5 signaling to TfR expression in erythropoiesis [317]. Also, IRP-2 knock out mice show disturbances of dopamine regulation as well as iron overload and increased ferritin expression in distinct brain areas and it is suggested that iron mismanagement upon loss of IRP-2 control accelerates the aging of dopaminergic neurons [318,319]. As stated above, IRP-2 is considered to be the main regulator of iron metabolism under normal conditions and may compensate for IRP-1 deficiency [320]. However, the responsiveness of IRP-1 and 2 to stress related stimuli, which may involve changes of the phosphorylation state, points at distinct roles of both IRPs in controlling the cellular labile iron pool under stress conditions.

### 3.4. NO Signaling and IRP Regulation

Nitric oxide synthase (NOS) generated nitric oxide (NO^●^) and peroxynitrite (ONOO^−^) derived thereof by reaction with superoxide (NO^●^ and ONOO^−^ representing reactive nitrogen species—RNS) are able to react with Fe contained in proteins as heme or Fe-S cluster bound iron [321,322,323,324,325,326,327]. Of note, NO^●^ which has a high affinity to iron [328] can mobilize iron from ferritin in a GSH dependent manner [329,330]. In addition, NO^●^ as well as the nitrosonium cation (NO^+^) can S-nitrosylate thiol groups of proteins including ferritin and IRPs which confers important regulatory functions in iron metabolism including changes of ferritin and TfR synthesis [328,331,332,333,334,335]. NO^●^ may also react with ferrous “labile” iron and thiol containing GSH which generates dinitrosyl-iron complexes [336] leading to S-nitrosothiol formation [337,338]. Of special relevance, nitrosylation of GSH by NO^+^ produces S-nitrosoglutathione (GSNO) [339,340,341,342,343] a potent antioxidant which exerts cytoprotective properties [341,344,345,346,347,348,349,350] albeit a hepatocytotoxic effect of GSNO has also been reported [351].

Several investigations have addressed the interference of NO-signaling with IRP-1 and 2 activity via NO^●^/ONOO^−^—Fe-S cluster interaction and IRP S-nitrosylation—which may reversibly (NO^+^) or irreversibly (ONOO^−^) inhibit IRP-1 aconitase activity, stabilize IRP-1 RNA binding (NO^●^) or irreversibly modify IRP-1 thus abrogating RNA binding (ONOO^−^) and also affect IRP-2 stability [311,325,332,333,352,353,354,355,356,357,358,359,360,361]. In particular, the enhanced degradation of IRP-2 mediated by NOS derived NO^●^ which triggers ferritin synthesis in cells exposed to proinflammatory stimuli points at the importance of NO-signaling in pathophysiological contexts [311,352,357]. However, it has been reported that NO^●^ can also stabilize IRP-2 probably by LIP interference [359,361,362] while IRP-2 degradation is promoted by NO^+^ mediated nitrosylation [333]. Notably, NO^+^ is able to stimulate ferritin synthesis also in an IRP-2 independent mode [311]. Hence, cellular iron homeodynamics is regulated by nitrogen species based on complex, feedback-regulated mechanisms, the expression of NOS and thus NO levels itself being directly affected by cellular iron levels [363]. Moreover, NO-signaling also allows an intercellular control of iron pools and by this may contribute to cell–cell interaction mediated changes of iron homeodynamics. Albeit is has been shown that macrophages stimulate iron release from target cells [364] it is questionable whether this is mediated by NO, however, evidence exists that NO can limit transferrin/TfR-based iron uptake (discussed in [365]).

### 3.5. Additional Regulatory Roles of IRPs

Recently, additional functions of IRPs have been identified which are more indirectly related to iron metabolism (reviewed in [366]). In particular, it has been shown that IRP-1 also acts as negative translational regulator of hypoxia-inducible factor 2α (HIF2α) [367]. This interference affects several downstream targets of HIF2α such as erythropoietin (EPO) expression and by this erythropoiesis and hepcidin expression [368,369,370,371,372] as well as transcriptional activation of Fpn and DMT-1 [373,374,375] in addition to the IRP-1/mRNA-based regulation. Since HIF2α, like HIF1α, also affects tumor progression and tumor stem cell function [376,377], IRP-1 could also play a role in tumorigenesis. Moreover, tumor cell proliferation is enhanced upon overexpression of IRP-2 [378], the oncogene c‑myc upregulating IRP-2 but repressing H-ferritin [379]. Thus, the role of IRPs may change in the course of neoplastic transformation serving the tumor growth associated reprogramming of iron metabolism [380] and cellular iron homeodynamics.

## 4. Iron Homeodynamics under Stress Conditions—A Distinct Role for Ferritin?

**Figure 2:**
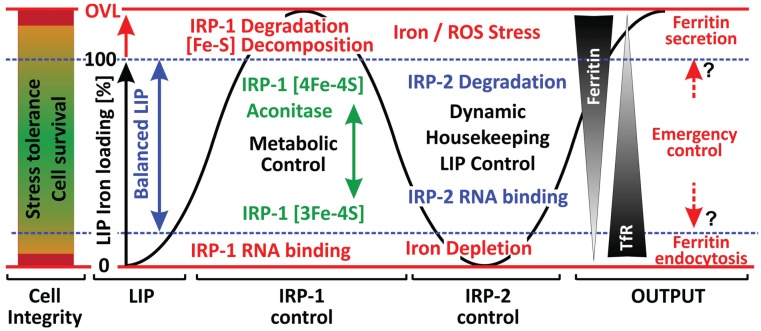
Iron homeodynamics and stress conditions. Cell integrity and stress tolerance demands a balanced LIP between 0 (iron depletion) and 100% (maximum loading). Under normal conditions (blue range) the LIP is controlled by IRP-2 abundance, IRP-1 preferentially exerting c-aconitase activity depending on Fe-S cluster conformation. Moderately enhanced oxidative stress will promote Fe-S cluster decomposition (see p. 820), while severe pro-oxidant regimens as well as iron overload (OVL) lead to IRP-1 degradation. Iron import via TfR and ferritin-based iron buffering control the LIP in opposite directions depending on the actual iron content and oxidant conditions (Output). It is hypothesized that ferritin exo- and endocytosis serve the “emergency control” of the LIP under conditions of either severe (or chronic) iron overload and oxidative stress or massive iron depletion, respectively.

Taken together, iron compartmentalization together with iron-, redox- and stress dependent gene expression (on the transcriptional and translational level) constitutes the framework of cellular iron homeodynamics. Transcriptional control of iron metabolism related genes defines a distinct *mRNA signature* [381] which is translated into an iron management-related proteome serving the dynamic fine-adjustment of intracellular iron balance. Stress conditions will modulate the cell type and condition (e.g., iron requirement, state of differentiation, proliferation) specific *mRNA signature* and even more specifically its translation, which is under IRP control. IRP activity by itself is directly (IRP-1) or indirectly (IRP-2) regulated by the LIP/CLIP, which involves regulatory feedback loops (e.g., MLIP dependent Fe-S cluster synthesis acting directly on IRP-1) as well as additional stress-related signals (e.g., NO-signaling). Hence, IRPs serve as central guardians of cellular iron homeodynamics and stress tolerance as illustrated in Figure 2.

Under normal conditions iron homeodynamics is predominantly determined by mitochondrial iron consumption (MLIP), IRP-2 serving the dynamic housekeeping adjustment of the LIP. Tightly coordinated with this, IRP-1 c-aconitase activity links the LIP to GSH and NADPH abundance and via this to cellular antioxidant capacity. Oxidative stress markedly interferes with this regulatory network depending on the source (ROS, RNS), severity and persistence of the pro-oxidant stressor. Moderate stress conditions promote Fe-S cluster disassembly and stimulate IRP-1 RNA binding which fosters iron overload. When antioxidant defenses are inadequate, this could aggravate the pro-oxidant condition especially with respect to the lysosomal and mitochondrial compartment. On the other hand, severe or chronic states of increased oxidative stress will lead to enhanced IRP degradation (IRP-1 and 2) which promotes iron (LIP) depletion due to elevated ferritin synthesis and reduced TfR-based iron import. Complicated by the concomitant decline of IRP-1 c-aconitase activity, which interrupts refueling of the antioxidant pools, this pro-oxidant condition readily will become incompatible with cell survival. Therefore, oxidative stress, especially when persistent, demands specific adaptations of iron management that support proper LIP control and continued iron supply for metabolic needs.

Moreover, severe ROS attack on iron-loaded ferritin could become a further considerable threat when the “safely stored iron” is liberated rendering the LIP (CLIP) uncontrollable due to an impaired iron buffering capacity. Hypothetically, ferritin/iron-rich cells such as hepatocytes and macrophages as well could face the risk of irreversible ferritin-iron/ROS derived damage by releasing iron loaded ferritin under oxidative stress which would rapidly lower the tenuous iron burden. The increase of serum ferritin associated with hepatic iron overload in ferroportin disease [55] provides support to this assumption. Similarly, in macrophages ferritin release could compensate the heme degradation-based iron charging of the CLIP/ferritin—a notion that fits with the finding that serum ferritin is mainly derived from macrophages [59]—which may increase under oxidative stress [382]. Furthermore, primary hepatocytes release ferritin *in vitro* in particular at initial culture stages [383,384], the secreted ferritin exerting an iron-dependent cytotoxic effect [95]. It cannot be excluded that this also reflects an attempt of the freshly isolated cells to mitigate the cell isolation derived pro-oxidant condition by emptying their intracellular iron buffer. On the contrary, it should not be neglected that serum ferritin may deliver at least 100 times more iron per molecule than transferrin. Thus, endocytosis of serum ferritin could counteract iron undersupply in cells with enhanced iron needs such as oligodendrocytes and erythroid precursor cells [24,63].

## 5. Conclusions

In conclusion, cellular iron homeodynamics is based on a well-orchestrated interaction of iron uptake, intracellular transport, iron storage, usage and export, which is embedded in cellular metabolic and surveillance control. Under normal conditions this machinery provides a dynamic response to changing iron requirements and iron supply allowing the constant fueling of intracellular iron metabolism. Under stress conditions, this orchestration changes in order to maintain homeodynamics and protect the cell from severe destabilization. Potentially, this may also involve distinct emergency control mechanism such as the release or uptake of ferritin to and from the extracellular environment, the possible existence of such alternative pathways remaining to be defined.

## Abbreviations

CLIP: cytosolic labile iron pool
DMT1: divalent metal transporter
ELC: endo-/lysosomal compartment
ELIP: endo-/lysosomal labile iron pool
Fpn: ferroportin
Ftx: frataxin
HNE: 4-hydroxy-nonenal
FRDA: Friedreich’s ataxia
IRE: iron regulatory elements
IRP: iron regulatory protein
ISCU: iron-sulfur cluster forming unit
LIP: labile iron pool
LMP: lysosomal membrane permeability
MLIP: mitochondrial labile iron pool
mtFER: mitochondrial ferritin
NTBI: non-transferrin bound iron
RME: receptor mediated endocytosis
RNS: reactive nitrogen species
ROS: reactive oxygen species
TBI: transferrin-bound iron
TfR: transferrin receptor
TRPML1: type IV mucolipidosis-associated protein
